# Dietary Effects, Age, and Urban–Rural Dynamics in Shaping Gut Microbiota of Elderly Vietnamese: A Cross-Sectional Study

**DOI:** 10.3390/microorganisms13122803

**Published:** 2025-12-09

**Authors:** Adel Hamdi, Le Van Truong, Vu Thi Hien, Phuong Anh Nguyen, Thi Bach Duong Hoang, Charmaine Lloyd, Rajaraman Eri, Dragana Stanley, Dong Van Quyen, Thi Thu Hao Van

**Affiliations:** 1 School of Science, RMIT University, Bundoora, VIC 3083, Australia; aao8537@hotmail.com (A.H.); charmaine.lloyd@rmit.edu.au (C.L.); rajaraman.eri@rmit.edu.au (R.E.); 2 Institute of Biology, Vietnam Academy of Science and Technology, Hanoi 10000, Vietnam; lvtruong@ib.ac.vn (L.V.T.); vuthihienibt@gmail.com (V.T.H.); dvquyen@ib.ac.vn (D.V.Q.); 3 Department of Life Sciences, University of Science and Technology of Hanoi, Vietnam Academy of Science and Technology, Hanoi 10000, Vietnam; phuonganhng148@gmail.com; 4 New Horizon Palliative Care Company Limited, Hanoi 10000, Vietnam; duong.hoangbach@gmail.com; 5 Central Queensland Innovation and Research Precinct (CQIRP), Institute for Future Farming Systems, Central Queensland University, Rockhampton, QLD 4701, Australia; d.stanley@cqu.edu.au

**Keywords:** gut microbiota, aging, diet, alpha diversity, beta diversity, 16S rRNA sequencing, healthy aging

## Abstract

Aging is associated with alterations in gut microbiota, yet the combined effects of geography and diet remain underexplored in elderly populations. This study investigated the gut microbiota of 227 healthy Vietnamese individuals aged ≥60 years, stratified by select urban and rural residence in both Hanoi and Thanh Hoa provinces, and across three age groups (60–69, 70–79, ≥80 years). Dietary patterns were collected and recorded for each participant. 16S rRNA gene sequencing revealed significant differences in microbial diversity and composition associated with geographical location (urban, rural) and age. Urban participants in Hanoi exhibited higher richness and greater abundance of health-associated genera, including *Bifidobacterium*, *Fusicatenibacter*, and *Blautia*, likely reflecting more diverse plant-based diets. In contrast, rural participants in Thanh Hoa showed enrichment of beneficial butyrate-producing genera such as *Fusicatenibacter*, *Roseburia*, *Lachnospira* and *Blautia*, possibly linked to traditional diets rich in freshwater fish and fermented foods. Participants aged 70–79 years displayed reduced microbial richness compared to other age groups. Age-related reductions in *Roseburia*, *Veillonella*, and *Prevotella* were also observed. These findings highlight how geography, diet, and aging shape the gut microbiota and may guide microbiota-targeted dietary strategies to promote healthy aging.

## 1. Introduction

The human gut microbiome is a diverse and dynamic ecosystem consisting of trillions of microbial cells, primarily bacteria, that reside predominantly in the colon [1,2,3,4]. This complex community plays a vital role in regulating digestion, metabolism, immune function, and host defense [5,6]. Disruptions to this ecosystem—referred to as dysbiosis—have been implicated in a variety of conditions, including inflammatory, metabolic, and neurodegenerative diseases [7,8,9,10,11].

Multiple host and environmental factors influence gut microbial composition such as age, diet, medications (especially antibiotics), physical activity, socioeconomic factors, immune status and overall well-being [7,12,13,14,15]. Among these determinants, diet is consistently identified as one of the strongest modifiable drivers of gut microbiome composition [16], while medications such as antibiotics [17] and proton-pump inhibitors [18] can exert dominant short-term effects. Long-term geographic and lifestyle exposures also shape population-level microbial profiles, particularly in older adults [19]. These variables have complex, interrelated effects on microbiota structure and function. Notably, aging is often accompanied by a reduction in microbial diversity and the depletion of beneficial bacteria, such as *Bifidobacterium*, alongside an increase in opportunistic taxa, including *Staphylococcus*, *Streptococcus*, *Enterococcus*, and *Enterobacteriaceae* [20]. These shifts may contribute to immunosenescence, frailty, chronic low-grade inflammation (“inflammaging”), and increased disease susceptibility [21,22,23,24]. However, some older individuals maintain microbiota profiles resembling those of younger adults, suggesting that diet and lifestyle may mitigate age-related microbial decline [25,26]. Recent evidence also shows that therapeutic interventions, such as direct-acting antivirals (DAAs), can reshape gut microbiota composition and diversity, reinforcing that environmental and lifestyle modifications, including diet, can modulate microbial recovery [27].

Centenarians are increasingly studied as models of healthy aging, having avoided many age-associated diseases [24,28,29]. Their gut microbiota often features beneficial taxa, particularly anti-inflammatory and short-chain fatty acid (SCFA) producers, that support gut barrier integrity, immune balance, and systemic metabolic health [30,31]. However, not all elderly populations exhibit the same microbial features, and variation may reflect broader cultural, dietary, and environmental exposures.

Although interest in aging and the microbiome is growing, data from Southeast Asia remain limited. Vietnam is particularly suited for microbiome-longevity research due to its high life expectancy, pronounced urban–rural divide and enduring traditional dietary patterns. Vietnamese centenarians typically consume rice-based, plant-forward diets, moderate amounts of animal protein, and fermented foods rich in natural probiotics [32,33,34]. These diets, along with physically active rural lifestyles, create unique environmental exposures that likely shape the aging gut microbiome [35]. Importantly, Vietnamese dietary habits differ substantially from other Asian cohorts, particularly in the types of herbs, vegetables, seafood, and region-specific fermented foods consumed [36]. Furthermore, Vietnam is undergoing one of the fastest nutrition and lifestyle transitions in Southeast Asia, creating a sharper contrast between traditional rural diets and increasingly processed urban dietary patterns compared with neighboring countries [37,38]. While it is difficult to control for all potential confounders in population-based studies, our study focuses on three interlinked determinants—age, diet, and urban–rural residence—within a relatively healthy elderly Vietnamese population. This approach allows for targeted exploration of microbial shifts in a culturally distinct and underrepresented setting.

Urbanization has emerged as a critical factor influencing microbiota composition. Urban lifestyles are often associated with processed foods, reduced fiber intake, higher antibiotic exposure, and more sedentary behavior, while rural populations tend to maintain traditional dietary patterns and active routines [39,40,41]. Thus, cross-sectional comparisons between rural and urban elderly can reveal how lifestyle and environment intersect with microbial aging trajectories [42,43,44].

Despite advances in sequencing technologies and growing interest in aging microbiome, its characterization remains challenging due to inter-individual variability and confounding factors such as medications, comorbidities, genetics, physical activity, environment and diet [45,46,47,48,49,50,51,52,53]. Moreover, reliance on Western cohorts limits the generalizability of findings to other regions. Observed differences in the microbiota among centenarians in Japan, Italy, and China suggest that cultural and dietary contexts play a critical role in shaping longevity-associated microbial signatures [30,54,55]. These insights may guide microbiome-targeted strategies, as recent evidence supports therapeutic modulation of gut microbiota to promote homeostasis in age-related conditions [56].

We hypothesized that elderly individuals living in rural Vietnamese settings, due to greater adherence to traditional diets and more natural environmental exposure, would exhibit higher microbial diversity and a greater abundance of health-associated taxa, including beneficial SCFA-producing genera [57], compared to their urban counterparts. However, the impact of rapid urbanization, dietary transitions, and evolving healthcare access may be reshaping these expected patterns.

This study aims to characterize the gut microbiome composition of healthy elderly Vietnamese individuals by comparing cohorts from urban and rural areas in two provinces (Hanoi and Thanh Hoa), and to investigate microbial differences associated with age and diet. Using 16S Ribosomal RNA (16S rRNA) gene sequencing, we assess microbial diversity, community structure, and differential abundance to explore how dietary habits and demographic factors influence the microbiota of aging adults.

## 2. Materials and Methods

### 2.1. Study Design and Sample Collection

This cross-sectional study was conducted across two provinces in Vietnam—Hanoi and Thanh Hoa, each comprising both urban and rural settings. A total of 227 self-reported healthy elderly participants aged 60 years or older were recruited through local community centers from four distinct cohorts: urban Hanoi (Thanh Xuan District, *n* = 47), rural Hanoi (Me Linh District, *n* = 69), urban Thanh Hoa (Dong Huong Ward, *n* = 56), and rural Thanh Hoa (Hoang Dai Ward, *n* = 55). Participants were stratified into three age groups: 60–69 years (*n* = 118), 70–79 years (*n* = 68), and ≥80 years (*n* = 41). In terms of gender distribution, 65 participants were male and 162 were female (Table 1). Inclusion criteria required participants to have regular bowel movements, no antibiotic use within four weeks prior to sample collection, and no use of medications that may affect gut microbiota, including antidiabetic drugs and proton pump inhibitors. Individuals with gastrointestinal disorders (e.g., chronic colitis, diarrhea, or constipation) or chronic diseases were excluded. Informed consent was obtained, and participants completed questionnaires detailing demographic information, diet (as described in Table 2 and Table 3) and general health. Stool samples were self-collected using sterile containers and stored on ice and transport to laboratory for processing. All samples were collected during the summer (June–August) to minimize seasonal variation in diet and microbiota composition. The study aimed to investigate differences in gut microbiota composition based on geographic location (urban vs. rural) and age group (60–69, 70–79, and ≥80 years) among healthy elderly individuals. The study was approved by the Institute of Genome Research Institutional Review Board for Biomedical Research, and all procedures were conducted in accordance with the relevant guidelines and regulations (Ethics approval number: 05-2-23/NCHG-HDDD).

### 2.2. Survey of Food Intake of Participants

To gain insights into the dietary habits of study participants, a structured food frequency questionnaire was administered to each individual at the time of sample collection. The survey form was designed to capture the habitual intake of key food groups, including vegetables, fruits, meats, fish, and fried or fast foods.

Participants were asked how frequently they consumed each food category, with options ranging from “three times or more per day” to “rarely.”. Special attention was paid to the frequency of fast-food consumption (e.g., pizza, fried chicken, French fries), which was categorized as: more than three times per week, two to three times per week, one time per week, two to three times per month, once per month, or rarely.

This dietary data was used to contextualize microbial differences between participants and contributed to the analysis of diet-associated microbial signatures across different age groups and geographic locations.

### 2.3. DNA Extraction and 16S rRNA Gene Amplification

Stool DNA was extracted using a validated protocol combining mechanical (bead beating), enzymatic (lysozyme, lysostaphin, mutanolysin), and chemical lysis with 4 M guanidine thiocyanate, 10% N-lauryl sarcosine, and 100 mg/mL polyvinylpolypyrrolidone (PVPP). DNA was precipitated with ethanol, treated with RNase A, and purified via sodium acetate. Reagents included EDTA, NaCl, sodium phosphate buffer, SDS, isopropanol, and ethanol, as described in previous work [58]. All chemicals were purchased from Sigma-Aldrich (St. Louis, MO, USA).

The bacterial 16S rRNA gene was amplified targeting the V3–V4 region using dual-index primers—pro341F (5′-CCTACGGGNBGCASCAG-3′) and pro805R (5′-GACTACNVGGGTATCTAATCC-3′), which incorporated index sequences, heterogeneity spacers, and Illumina adapter linkers (Illumina, San Diego, CA, USA) [59].

PCR reactions were performed in a final volume of 20 µL using Q5 High-Fidelity 2× Master Mix (New England Biolabs, Ipswich, MA, USA), 250 nM of each primer, and 1 µL of template DNA. Amplification was carried out on an Eppendorf Mastercycler Pro (Eppendorf AG, Hamburg, Germany) under the following conditions: initial denaturation at 98 °C for 1 min; 35 cycles of 98 °C for 10 s, 49 °C for 30 s, and 72 °C for 30 s; followed by a final extension at 72 °C for 10 min. Following amplification, PCR products were visualized by agarose gel electrophoresis to confirm the presence and size of the expected amplicon bands prior to sequencing. Negative controls (nuclease-free water) were included and showed no amplification.

### 2.4. Sequencing and Bioinformatics Analysis

Amplicon sequencing was performed using the Illumina MiSeq platform with 2 × 300 bp paired-end reads. Raw sequences were trimmed using Trimmomatic v0.39 to remove low-quality reads and adapters [59]. Denoising, Quality filtering, chimera removal and feature inference were conducted using the Divisive Amplicon Denoising Algorithm 2 (DADA2) pipeline within Quantitative Insights Into Microbial Ecology 2 (QIIME2) v2020.6 [60,61], producing high-resolution ASVs. Taxonomic classification was assigned using the SILVA rRNA Database (v138) [62].

### 2.5. Statistical and Diversity Analyses

Microbial community profiling and statistical analyses were performed using MicrobiomeAnalyst (https://www.microbiomeanalyst.ca/MicrobiomeAnalyst/home.xhtml, accessed on 2 December 2025) [60]. Alpha diversity (Chao1, Shannon) was calculated from rarefied ASV tables. Non-parametric tests were applied: Mann–Whitney for two-group and Kruskal–Wallis for multi-group comparisons, with post hoc pairwise testing and FDR correction (Benjamini–Hochberg). Beta diversity was assessed using Bray–Curtis dissimilarity, visualized by PCoA, and tested with PERMANOVA. Differentially abundant taxa across groups were identified using LEfSe and single factor implemented in the MicrobiomeAnalyst software with FDR-adjusted *p*-values and an LDA score threshold of ±2.0. Prior to differential abundance testing, relative abundance values were log-transformed using base-10 logarithm (log10), following MicrobiomeAnalyst’s default settings.

To standardize abundance values prior to differential abundance analysis and visualization, MicrobiomeAnalyst applies total-sum scaling (TSS), converting counts to relative abundances and multiplying by the platform’s default scaling factor. These TSS-scaled abundances were subsequently log10(x + 1)-transformed following MicrobiomeAnalyst’s default settings. All reported *p*-values are FDR-corrected.

## 3. Results

### 3.1. Gut Microbiome Profiling: Sequencing Depth and Data Quality

Following quality control, a total of 2,178,830 high-quality paired-end reads were obtained, with an average of 9598 reads per sample. To reduce noise and minimize the influence of sequencing artifacts, the Amplicon Sequence Variants (ASVs) table was filtered to exclude features present in only a single sample and those with a total abundance of less than 100 reads. The number of features that remained after the data filtering step is 5163 for further investigation. The raw sequence data generated in this study was submitted to the NCBI SRA database and are publicly available under accession number PRJNA1331965.

### 3.2. Diet Diversity and Regional Eating Patterns Among Elderly Vietnamese

Dietary data revealed notable regional and lifestyle-related differences. In Hanoi, vegetable intake was more frequent among urban participants, with 40.4% and 57.4% consuming vegetables three and twice daily, respectively, and none reporting rare consumption. In contrast, vegetable intake was lower in rural Hanoi, with only 18% consuming 3 times per day, 42.0% consuming twice daily, and 14.5% reporting rare intake. Similarly, fruit intake was more frequent in urban Hanoi (44.7% twice daily) compared to 27.5% in rural Hanoi, and rare consumption was much higher in rural Hanoi (30.4%) compared to urban areas (4.3%). Meat consumption patterns were broadly similar across groups, although rare intake was more common among rural participants (17.4%) than in urban participants (6.4%). Daily fish intake was more prevalent in urban Hanoi (42.6% vs. 17.4%, 2 twice daily), whereas more rural participants reported rarely consuming fish (42.0% vs. 6.4%). Fried food consumption showed little difference between the groups, with daily intake reported by 31.9% of urban and 27.5% of rural participants, and rare intake by 57.4% and 60.9%, respectively. Interestingly, fast food consumption showed the opposite trend—rare intake was more frequent in urban Hanoi (78.7%) compared to rural Hanoi (33.3%), and a greater proportion in rural Hanoi reported consumption ≥ 3 times/week (8.7% vs. 4.3%) (Table 2).

**Table 2:** Frequency (%) of vegetables, fruit, meat, fish, and fried food and fast food consumption among elderly participants in urban and rural Hanoi.

Food Group	Frequency	Urban Hanoi (%)	Rural Hanoi (%)
Vegetables	≥3/day	40.4	18.8
	2/day	57.4	42.0
	1/day	2.1	24.6
	Rarely	0.0	14.5
Fruits	≥3/day	27.7	11.6
	2/day	44.7	27.5
	1/day	23.4	30.4
	Rarely	4.3	30.4
Meat	≥3/day	10.6	13.0
	2/day	51.1	43.5
	1/day	31.9	26.1
	Rarely	6.4	17.4
Fish	≥3/day	4.3	7.2
	2/day	46.8	17.4
	1/day	42.6	33.3
	Rarely	6.4	42.0
Fried foods	≥3/day	2.1	4.3
	2/day	8.5	7.2
	1/day	31.9	27.5
	Rarely	57.4	60.9
Fast food	≥3 times/week	4.3	8.7
	2–3 times/week	2.1	8.7
	1 time/week	6.4	17.4
	2–3 times/month	6.4	17.4
	1 time/month	2.1	14.5
	Rarely	78.7	33.3

In Thanh Hoa province, dietary habits were more consistent between urban and rural groups. Nearly all participants consumed vegetables at least twice daily (89.3% urban vs. 96.4% rural), and none reported rare intake. Rare fruit consumption was notably higher in rural Thanh Hoa (30.9%) compared to none in urban areas. Daily fruit intake was also higher among urban participants (92.9%) than rural participants (69.1%). A quarter of rural Thanh Hoa participants rarely consume meat, compared with only 7.1% of urban Thanh Hoa participants. However, rural participants consume fish more frequently than the urban group, with 60.0% eating fish twice daily, compared to just 19.6% of urban participants. Both groups reported infrequent consumption of fried and fast food, with over 90% in each group indicating rare intake. No participants in either urban or rural Thanh Hoa reported fast food consumption two or more times per week (Table 3).

**Table 3:** Frequency (%) of vegetables, fruit, meat, fish, fried food and fast food consumption among elderly participants in urban and rural Thanh Hoa.

Food Group	Frequency	Urban Thanh Hoa (%)	Rural Thanh Hoa (%)
Vegetables	≥3/day	5.4	1.8
	2/day	89.3	96.4
	1/day	5.4	1.8
	Rarely	0.0	0.0
Fruits	≥3/day	7.1	0.0
	2/day	37.5	30.9
	1/day	55.4	38.2
	Rarely	0.0	30.9
Meat	≥3/day	5.4	0.0
	2/day	33.9	45.5
	1/day	53.6	29.1
	Rarely	7.1	25.5
Fish	≥3/day	0.0	1.8
	2/day	19.6	60.0
	1/day	48.2	18.2
	Rarely	32.1	20.0
Fried foods	≥3/day	1.8	0.0
	2/day	0.0	0.0
	1/day	5.4	9.1
	Rarely	92.9	90.1
Fast food	≥3 times/week	0.0	0.0
	2–3 times/week	0.0	0.0
	1 time/week	3.6	0.0
	2–3 times/month	1.8	3.6
	1 time/month	10.7	1.8
	Rarely	83.9	94.5

### 3.3. Alpha Diversity Patterns Differ by Region and Age in Vietnamese Elderly

Alpha diversity was assessed using the Chao1 and Shannon indices at the genus level to compare microbial richness and diversity across urban and rural populations in both Hanoi and Thanh Hoa, as well as among different elderly age groups. In Hanoi, microbial richness (Chao1) was significantly higher in urban participants compared to rural counterparts (*p* = 0.019), while no significant difference was detected in diversity (Shannon index; *p* = 0.201). In Thanh Hoa, no significant differences were observed between urban and rural groups for either richness (Chao1; *p* = 0.052) or diversity (Shannon; *p* = 0.054).

**Figure 1:**
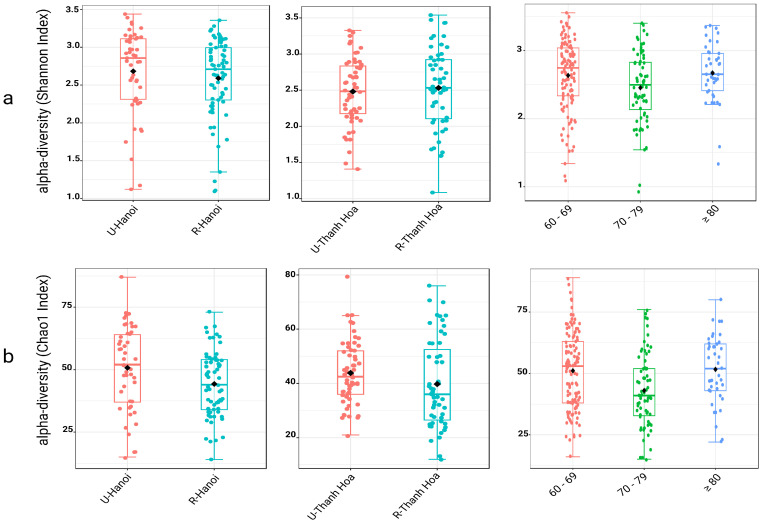
Alpha diversity of gut microbiota across location and age groups, assessed using two indices. Alpha diversity was evaluated using the Shannon index (**a**) to represent microbial diversity and the Chao1 index (**b**) to reflect microbial richness, both at the genus level. Each set of boxplots compares diversity between urban (U) and rural (R) populations in Hanoi (U-Hanoi vs. R-Hanoi), Thanh Hoa (U-Thanh Hoa vs. R-Thanh Hoa), and among different age groups (60–69, 70–79, and ≥80 years). Boxplots display the median, interquartile range, individual sample values. The black rhombuses represent the group means.

When analyzed by age, a non-linear pattern emerged. Participants aged 70–79 exhibited significantly lower richness and diversity than both younger (60–69) and older (≥80) groups. The Chao1 index showed significantly reduced richness in the 70–79 group compared to 60–69 (*p* = 0.003) and ≥80 (*p* = 0.003), with no significant difference between the 60–69 and ≥80 groups (*p* = 0.670). Similarly, the Shannon index revealed significantly lower diversity in the 70–79 group relative to the 60–69 (*p* = 0.04) and ≥80 groups (*p* = 0.04), while the latter two did not differ significantly (*p* = 0.98). These results highlight a dip in gut microbial richness and diversity specifically in the 70–79 age cohort, indicating a transient reduction in microbiome complexity with advancing age (Figure 1, Table S1).

### 3.4. Beta Diversity Across Regions and Age Groups

Beta diversity was assessed using Principal Coordinate Analysis (PCoA) based on Bray–Curtis dissimilarity to evaluate and visualize similarities/dissimilarities in the overall microbial community composition between urban and rural participants within each location and across age groups. In Hanoi, the PCoA plot demonstrated substantial overlap between U-Hanoi and R-Hanoi groups, and Permutational Multivariate Analysis of Variance (PERMANOVA) analysis confirmed no statistically significant differences (R^2^ = 0.01, *p* = 0.197), indicating similar overall microbial structures. In contrast, microbial community composition in Thanh Hoa showed clearer separation between U-Thanh Hoa and R-Thanh Hoa participants. PERMANOVA analysis supported these observations, revealing significant differences in beta diversity (R^2^ = 0.0545, *p* = 0.001).

When analyzed by age, PCoA revealed partial separation among the three age groups (60–69, 70–79, and ≥80 years). PERMANOVA analysis confirmed that overall community composition differed significantly across age groups (R^2^ = 0.0219, *p* = 0.003). Pairwise comparisons indicated significant differences between the 70–79 and ≥80 groups (R^2^ = 0.022, *p* = 0.01), between the ≥80 and 60–69 groups (R^2^ = 0.022, *p* = 0.003) and between the 70–79 and 60–69 groups (R^2^ = 0.008, *p* = 0.082) (Figure S1, Table S2).

### 3.5. Location- and Age-Associated Shifts in the Relative Abundance of Dominant Gut Microbial Genera

The relative abundance of the top 20 gut microbial genera revealed distinct patterns across location and age groups among elderly participants. In Hanoi, *Bacteroides* was the dominant genus in both urban (16%) and rural (17%) groups. *Akkermansia* was more abundant in urban participants (1.9%) compared to rural (0.8%), and *Bifidobacterium* also showed higher levels in the urban group (3.3%) than in the rural group (0.7%), highlighting location-related microbial variation. In Thanh Hoa, *Bacteroides*, *Fusicatenibacter*, and *Roseburia* were more abundant in rural participants (14.3%, 3.19%, and 1.9%, respectively) than in urban participants (5.5%, 0.5%, and 0.2%, respectively), while *Collinsella* showed higher levels in the urban group (6.1%) compared to the rural group (1.0%). No notable differences in *Bifidobacterium* were observed between groups (Supplementary Figure S2).

Age-associated trends were also evident, with *Bacteroides* increasing progressively with age, reaching the highest relative abundance in the ≥80 group (20.1%), while *Prevotella* declined from 12.8% in the 60–69 group to 8.9% in the ≥80 group. *Collinsella* exhibited a marked increase in the oldest group (4.1%). *Agathobacter* was more abundant in the 70–79 group. No notable differences in *Bifidobacterium* were observed across age groups (Figure S3, Tables S3–S5).

### 3.6. Univariate Analysis of Differentially Abundant Genera Between Urban and Rural Groups

**Figure 2:**
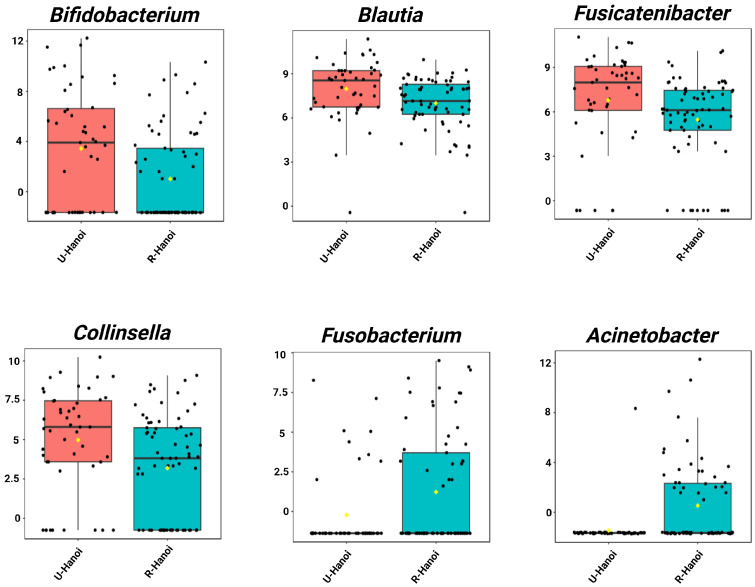
Single-factor analysis of differentially abundant genera between urban (U-Hanoi, red) and rural (R-Hanoi, blue) participants in Hanoi. Genera with LDA scores ± 2.0 and *p* < 0.05 are shown. Values represent TSS-scaled relative abundances log10(x + 1)-transformed. The yellow rhombuses represent the group means.

Single-factor analysis revealed distinct genera with significantly different relative abundances between urban and rural elderly participants in both Hanoi and Thanh Hoa (LDA score > 2.0, *p* < 0.05). In Hanoi, eight genera were identified as significantly different. The urban group (U-Hanoi) showed higher abundances of *Bifidobacterium*, *Fusicatenibacter*, *Collinsella*, and *Blautia*. In contrast, the rural group (R-Hanoi) was enriched with *Fusobacterium* and *Acinetobacter* (Figure 2, Table S7).

**Figure 3:**
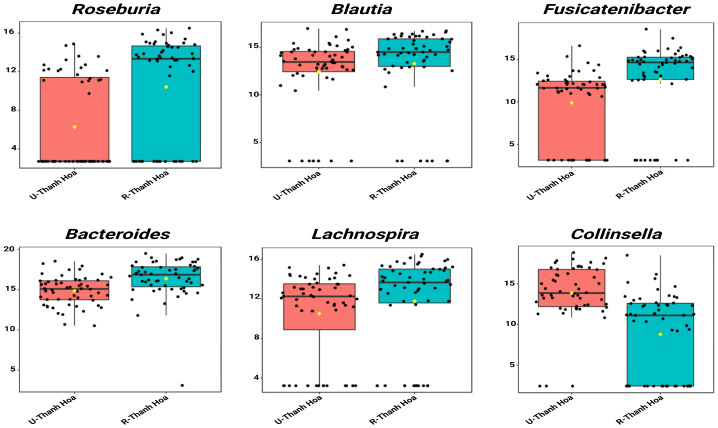
Single-factor analysis of differentially abundant genera between urban (U-Thanh Hoa, red) and rural (R-Thanh Hoa, blue) participants in Thanh Hoa. Genera with LDA scores ± 2.0 and *p* < 0.05 are shown. Values represent TSS-scaled relative abundances log10(x + 1)-transformed. The yellow rhombuses represent the group means.

In Thanh Hoa, single-factor analysis identified 6 genera that differed significantly between urban (U-Thanh Hoa) and rural (R-Thanh Hoa) participants. Urban individuals had an increased abundance of *Collinsella.* Conversely, the rural group showed enrichment in *Fusicatenibacte*, *Bacteroides*, *Lachnospira*, *Roseburia* and *Blautia* (Figure 3, Table S8).

### 3.7. Univariate Analysis of Age-Associated Differences in Gut Microbial Genera

LEfSe analysis identified the top 15 genera with significantly different relative abundances across the three elderly age groups (60–69, 70–79, and ≥80 years). The ≥80 age group was characterized by a higher abundance of *Collinsella*, *Butyrivibrio*, *Senegalimassilia*, *Bacteroides_pectinophilus*_group, *UCG_005*, *lachnospiraceae*_NK4A136_group, *Bacillus* and *Cerasibacillus*. In the 70–79 age group, *Ruminococcus*_gnavus_group was enriched. Meanwhile, the 60–69 group exhibited increased levels of *Eubacterium eligens* group, *Roseburia*, *Butyricicoccus* and *Veillonella* (Figure S4, Table S6).

**Figure 4:**
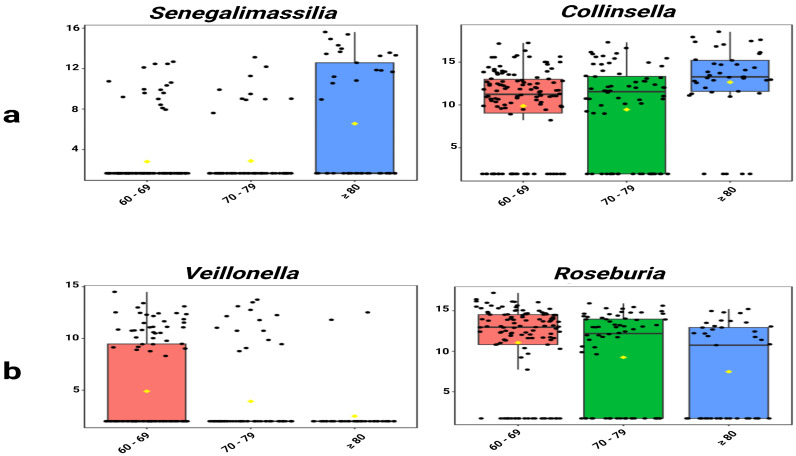
Age-associated trends in selected genera. (**a**) *Senegalimassilia* and *Collinsella* showed significantly increasing abundance with age. (**b**) *Veillonella* and *Roseburia* showed significantly decreasing abundance. Values represent TSS-scaled relative abundances log10(x + 1)-transformed. The yellow rhombuses represent the group means.

Single-factor analysis revealed distinct age-specific enrichment patterns. *Senegalimassilia* (*p* = 0.001) and *Collinsella* (*p* = 0.014) showed significantly increasing abundance with age (Figure 4a). Conversely, *Veillonella* (*p* = 0.036) and *Roseburia* (*p* = 0.016) exhibited significantly decreasing trends (Figure 4b, Table S9).

## 4. Discussion

We initially hypothesized that rural participants would exhibit higher gut microbial diversity due to greater environmental exposure and diets richer in fiber and fermented foods [63,64,65,66], factors previously linked to better physical capacity and lower biological age [67,68]. Contrary to this expectation, urban Hanoi participants displayed significantly greater microbial richness than their rural counterparts. This aligns with recent evidence showing no consistent association between diversity and chronological or biological age, nor with physical function [69].

In this study, urban Hanoi participants reported higher daily intake of vegetables (≥2/day: 97.8% vs. 60.8%) and fruits (≥2/day: 72.4% vs. 39.1%) than their rural counterparts, which is contrary to our initial assumption and may explain their greater microbial richness. These dietary patterns corresponded with a higher abundance of health-associated genera such as *Bifidobacterium*, *Fusicatenibacter*, and *Blautia*, which are known for their immunomodulatory effects, SCFA production and maintenance of gut barrier integrity [70,71,72,73,74,75,76]. Similar enrichment of *Bacteroides* and *Blautia* has also been observed in Japanese and Korean elderly cohorts residing in highly urbanized areas like Kyoto and Seoul [77,78,79]. In contrast, rural participants had lower intake of plant-based foods—evidenced by higher rates of rare vegetable (14.5% vs. 0%) and fruit intake (30.4% vs. 4.3%)— alongside reduced fish consumption (rarely: 42.0% vs. 6.4%) and greater fast-food intake (66.7% consuming fast food ≥ 1 time/month vs. 21.3% in urban Hanoi) (Table 2). These patterns corresponded with the enrichment of potentially pro-inflammatory and opportunistic pathogens like *Fusobacterium* and *Acinetobacter* [80,81,82,83,84]. This suggests that plant-based dietary diversity and fish intake support healthier gut microbiota, while low-fiber, processed-food-rich diets may promote harmful bacteria [85,86,87,88]. Despite our hypothesis anticipating the opposite, urban dwellers may benefit from increased exposure to health education, preventative care, and access to nutritional guidance, which could lead to dietary habits and lifestyles more conducive to maintaining microbial diversity and resilience.

In contrast, rural participants in Thanh Hoa exhibited a higher relative abundance of beneficial genera, including *Fusicatenibacter*, *Lachnospira*, *Roseburia*, and *Blautia* [74,75,89]. This profile may be partially attributed to the significantly greater consumption of fish in the rural cohort (≥2/day: 61.8% vs. 19.6%), which provides omega-3 fatty acids known to promote anti-inflammatory and butyrate-producing bacteria [88]. Additionally, rural participants reported lower fast-food intake (rarely: 94.5% vs. 83.9%), potentially limiting dietary sources of dysbiosis-associated taxa. Although a higher proportion of rural participants reported rare fruit consumption (30.9% vs. 0% in urban), this may reflect seasonal availability in rural areas rather than an absence of plant-based diversity. Traditional dietary practices likely persist in these communities, with a strong reliance on home-prepared meals based on rice, vegetables, freshwater fish, and fermented products such as soybean sauce paste (Tuong), shrimp paste (Tom chua), fermented rice (Com me) and fish-based condiments such as fermented fish paste (Mam chua) and sauce (Nuoc mam) [34]. These fermented foods are rich in lactic acid bacteria and microbial metabolites that may further support the growth of saccharolytic and butyrate-producing taxa such as *Roseburia* [90]. Notably, *Roseburia* and *Lachnospira* were also enriched in rural Japanese and Korean elderly populations from longevity villages [77,79], where fermentation-based diets are prevalent [91,92]. Collectively, these findings highlight how local dietary patterns and cultural traditions in rural Thanh Hoa can shape the gut microbiota in beneficial ways, underscoring the region-specific impact of urbanization on microbial ecology.

*Collinsella* showed consistent enrichment in urban populations of both provinces, echoing findings from urban Makassar, Indonesia [93], but contrasting with its rural dominance in China [94], suggesting a context-dependent distribution. Its abundance has been associated with low fiber intake [95,96], omnivorous diets [97], and ghee consumption [98], while decreasing under low-carbohydrate diets [48]. Although reduced in IBD [94], *Collinsella* has also been implicated in rheumatoid arthritis, hypercholesterolemia [98], symptomatic atherosclerosis [50], and certain cancers [99], indicating potential pathogenic roles in some contexts. The elevated levels of *Collinsella* observed among urban participants may be partially attributable to dietary patterns [95,100], particularly higher meat consumption, which was notably more frequent in urban cohorts across both Hanoi and Thanh Hoa (≥2/day) (Table 2 and Table 3). This suggests that urban dietary habits may contribute to the enrichment of *Collinsella*.

The long-standing view that gut microbial diversity declines progressively with age [101,102] has been increasingly contested. In our study, although alpha diversity—measured by Chao1 and Shannon indices—was reduced in individuals aged 70–79, it was notably higher in the oldest group (≥80 years). Similar trends have been reported in nonagenarians and centenarians, who often display greater microbial richness than younger elderly cohorts [103]. Kong et al. (2019) similarly observed elevated diversity in long-lived individuals from Jiangsu and Sichuan provinces in China [43]. Furthermore, no significant difference was found between the youngest elderly group (60–69 years) and the ≥80 cohort, aligning with a large Chinese population-based study showing that elderly microbiota profiles often resemble those of younger adults [26]. These findings suggest that gut microbial diversity does not decline in a linear trajectory with age; rather, a stabilization or even recovery of diversity may occur in longevity-associated populations. However, our data cannot conclusively rule on age-related decline, given the restricted age range studied (aged ≥60 years).

*Roseburia* and *Veillonella* were among the most frequently reported genera associated with age-related microbial changes. In our study, both genera declined significantly with increasing age. The reduction in *Roseburia* aligns with findings from Italian, Irish, and Thai cohorts [25,54,104,105], though opposite trends have been observed in Russian, Chinese, and Korean populations [106,107,108,109]. As a key butyrate producer, *Roseburia* supports gut homeostasis by downregulating pro-inflammatory cytokines Interferon-gamma (IFN-γ), Tumor Necrosis Factor-alpha (TNF-α), Interleukin-1 beta (IL-1β), Interleukin-6 (IL-6), Interleukin-8 (IL-8), upregulating anti-inflammatory mediators Interleukin-10 (IL-10) and Transforming Growth Factor-beta (TGF-β), and strengthening barrier integrity through mucin 2 and tight junction proteins [110]. Notably, both *Roseburia* and *Prevotella* are reduced in frail older adults, suggesting a link between their depletion and aging-related frailty [110,111,112,113]. Similarly, *Veillonella* abundance declined with age, consistent with findings in adults ≥ 75 years [114], though not replicated in all cohorts [108,115,116]. *Veillonella* metabolizes exercise-derived lactate into propionate and is enriched in physically active individuals, including marathon runners [117]. Its decline may therefore reflect reduced physical activity and lactate availability in older adults, supporting its potential as a biomarker of physical decline [114].

In this study, the genus *Senegalimassilia* exhibited a significant increase across age cohorts. This pattern may be partially influenced by gender distribution (Table 1). A previous study reported slightly higher *Senegalimassilia* abundance in females and also suggested a potential link with body weight [118]. Thus, the age-related increase observed in our cohorts may reflect, at least in part, the higher proportion of females in our cohorts. Future studies should adjust for sex and body mass index when examining microbial trends across aging populations to better disentangle age-specific effects from potential confounders.

This study offers new insights into the gut microbiota of elderly populations in Vietnam, demonstrating that both geographical location and age significantly influence microbial diversity and composition. Urban residents, particularly those in Hanoi, exhibited greater microbial richness and higher abundance of health-associated genera, likely shaped by diverse plant-based diets and better access to healthcare and nutritional education. In contrast, the opposite trend was observed in rural Thanh Hoa, were enriched in butyrate-producing genera such as *Fusicatenibacter*, *Roseburia*, and *Blautia*, reflecting traditional dietary patterns rich in freshwater fish and fermented foods. Age-stratified analysis revealed that microbial diversity does not decline uniformly with age; notably, individuals aged ≥80 years exhibited signs of diversity stabilization or recovery, accompanied by compositional shifts in key taxa including *Roseburia* and *Veillonella*.

Importantly, these findings also suggest translational potential, as microbiome-informed dietary strategies, such as increasing the intake of fiber-rich and fermented foods, could support gut health and improve aging outcomes among the Vietnamese elderly. Although some dietary differences were observed, overall dietary patterns aligned closely with regional residence, and the single-season sampling limited our ability to analyze diet as an independent factor. This study is also constrained by the absence of metabolomic data and longitudinal follow-up, which restricts functional interpretation. Future studies incorporating multi-season sampling, diverse dietary profiles, and long-term metagenomics are needed to clarify mechanisms and identify microbial markers that support healthy aging.

## Figures and Tables

**Table 1:** Distribution of study participants (*n* = 227) by age group and gender across urban and rural areas in Hanoi and Thanh Hoa.

	Age 60–69 (*n*)	Age 70–79 (*n*)	Age ≥ 80 (*n*)	Male (*n*)	Female (*n*)
Urban Hanoi (47)	19	22	6	10	37
Rural Hanoi (69)	66	3	0	15	54
Urban Thanh Hoa (56)	15	14	27	22	34
Rural Thanh Hoa (55)	18	29	8	18	37
Total (Male:Female)	118 (34:84)	68 (18:50)	41 (13:28)		

## Data Availability

The data presented in this study are openly available in NCBI SRA database at https://www.ncbi.nlm.nih.gov/search/all/?term=PRJNA1331965 with accession number PRJNA1331965.

## Supplementary Materials

The following supporting information can be downloaded at: https://www.mdpi.com/article/10.3390/microorganisms13122803/s1. Figure S1: Beta diversity of gut microbiota across location and age groups, visualized using PCoA; Figure S2: Relative abundance of the top 20 bacterial genera in urban and rural elderly populations from two Vietnamese provinces; Figure S3: Genus-level composition of gut microbiota across different elderly age groups in both provinces; Figure S4: LEfSe analysis of differentially abundant genera across age groups (60–69 years, 70–79 years, and ≥80 years). Table S1: Statistical results for alpha diversity (Chao1 and Shannon indices) across regions and age groups. Table S2: Statistical results for beta diversity (Bray–Curtis distances and PERMANOVA) across regions and age groups. Table S3: Genus-level relative abundance (%) of dominant taxa in urban and rural Hanoi. Table S4: Genus-level relative abundance (%) of dominant taxa in urban and rural Thanh Hoa. Table S5: Genus-level relative abundance (%) of dominant taxa across age groups (60–69, 70–79, and ≥79 years). Table S6: LEfSe-identified discriminative genera across age groups with corresponding LDA scores and relative abundance values. Table S7: Significant genus-level features from single-factor analysis comparing urban and rural Hanoi. Table S8: Significant genus-level features from single-factor analysis comparing urban and rural Thanh Hoa. Table S9: Significant genus-level trends from single-factor analysis across age groups (60–69, 70–79, and ≥79 years).

## Abbreviations

The following abbreviations are used in this manuscript:

SCFA	Short-Chain Fatty acid
16S rRNA ASV	16S Ribosomal RNA Amplicon Sequence Variant
OTUs	Operational Taxonomic Units
PCoA	Principal Coordinates Analysis
PERMANOVA	Permutational Multivariate Analysis of Variance
LEfSe	Linear Discriminant Analysis Effect Size
LDA	Linear Discriminant Analysis
IBD	Inflammatory Bowel Disease
PHC	Primary Health Care
V3–V4	Variable Regions 3 and 4 of the 16S rRNA Gene
DNA	Deoxyribonucleic Acid
MiSeq	Illumina MiSeq Sequencing Platform
DADA2	Divisive Amplicon Denoising Algorithm 2
QIIME2	Quantitative Insights Into Microbial Ecology 2
SILVA	SILVA rRNA Database
FDR	False Discovery Rate
IL-10	Interleukin-10
IFN-γ	Interferon-gamma
TNF-α	Tumor Necrosis Factor-alpha
IL-1β	Interleukin-1 beta
IL-6	Interleukin-6
IL-8	Interleukin-8
TGF-β	Transforming Growth Factor-beta
DAAs	Direct-acting antivirals
PVPP	polyvinylpolypyrrolidone
