# Supplementary material for: Dietary Effects, Age, and Urban–Rural Dynamics in Shaping Gut Microbiota of Elderly Vietnamese: A Cross-Sectional Study
Source: Microorganisms. 2025 Dec 9;13(12):2803. doi: 10.3390/microorganisms13122803 (PMC12735460; doi:10.3390/microorganisms13122803)
Supplement: Supplementary file 1 [file microorganisms-13-02803-s001.zip › microorganisms-3957723-supplementary.pdf]

microorganisms

MDPI

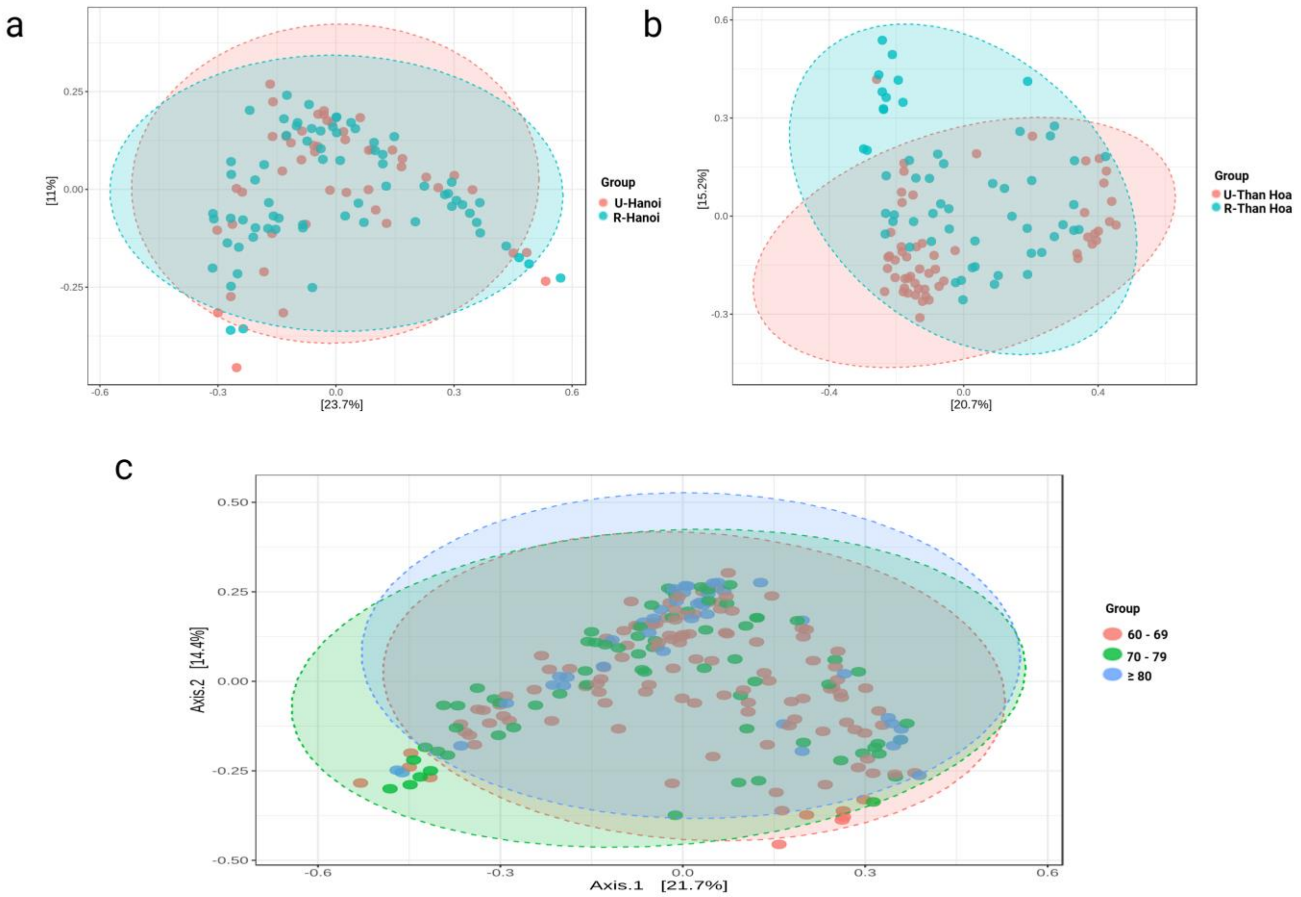

Figure S1. Beta diversity of gut microbiota across location and age groups, visualized using PCoA. Beta diversity was assessed using PCoA plots based on Bray–Curtis distance to compare microbial community composition between groups. Each panel displays comparisons between urban and rural populations in Hanoi (**a**), Thanh Hoa (**b**), and among age groups 60–69, 70–79, and ≥80 years (**c**). Each dot represents an individual's gut microbiota profile, and ellipses indicate 95% confidence intervals for group clustering. The percentage of variance explained by the first two principal coordinates is shown on the axes.

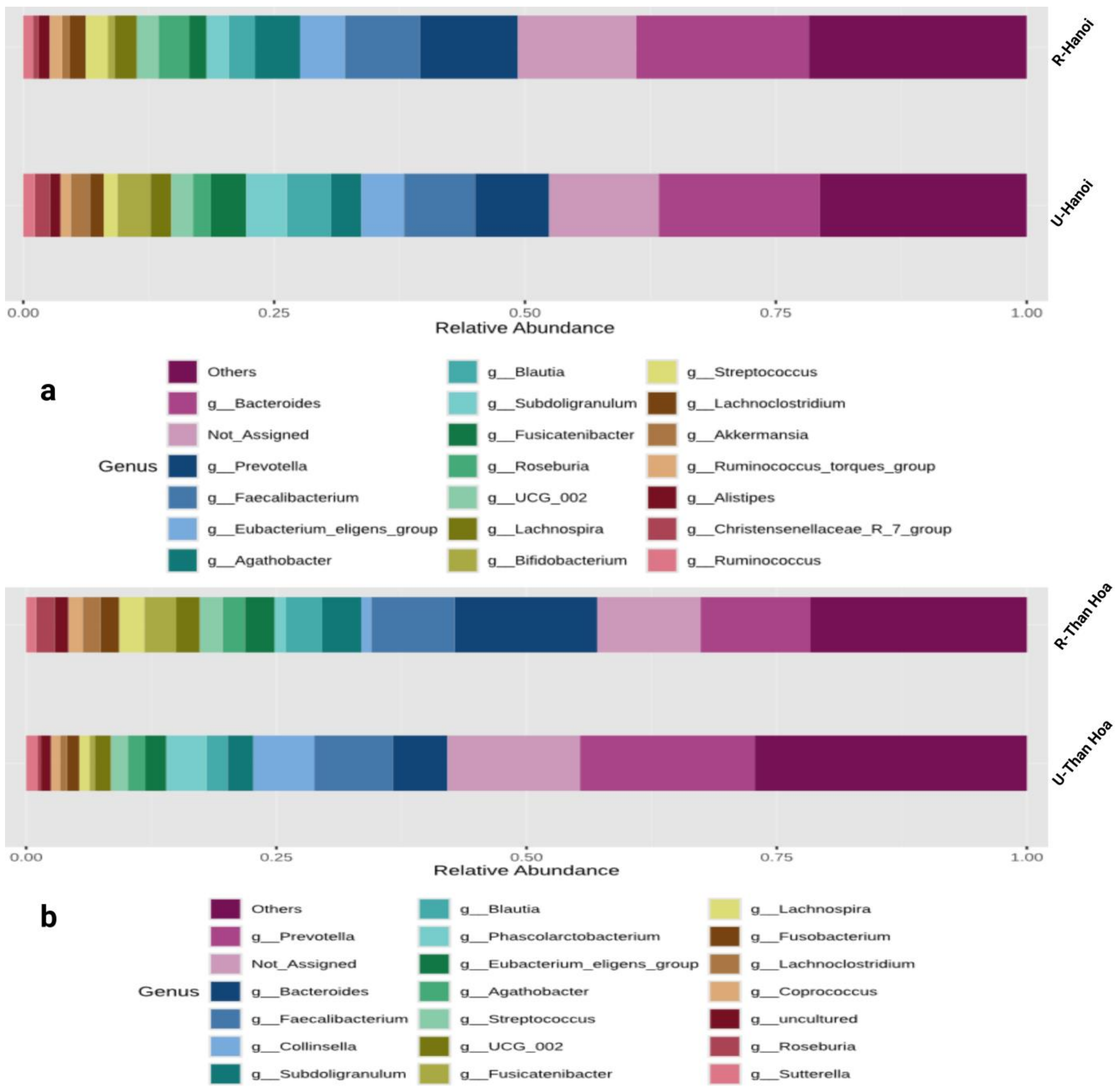

Figure S2. Relative abundance of the top 20 bacterial genera in urban and rural elderly populations from two Vietnamese provinces. (**a**) Genus-level composition of gut microbiota in urban (U-Hanoi) and rural (R-Hanoi) participants from Hanoi. (**b**) Genus-level composition in urban (U-Thanh Hoa) and rural (R-Thanh Hoa) participants from Thanh Hoa. Stacked bar plots illustrate the average relative abundance of the 20 most dominant genera in each group.

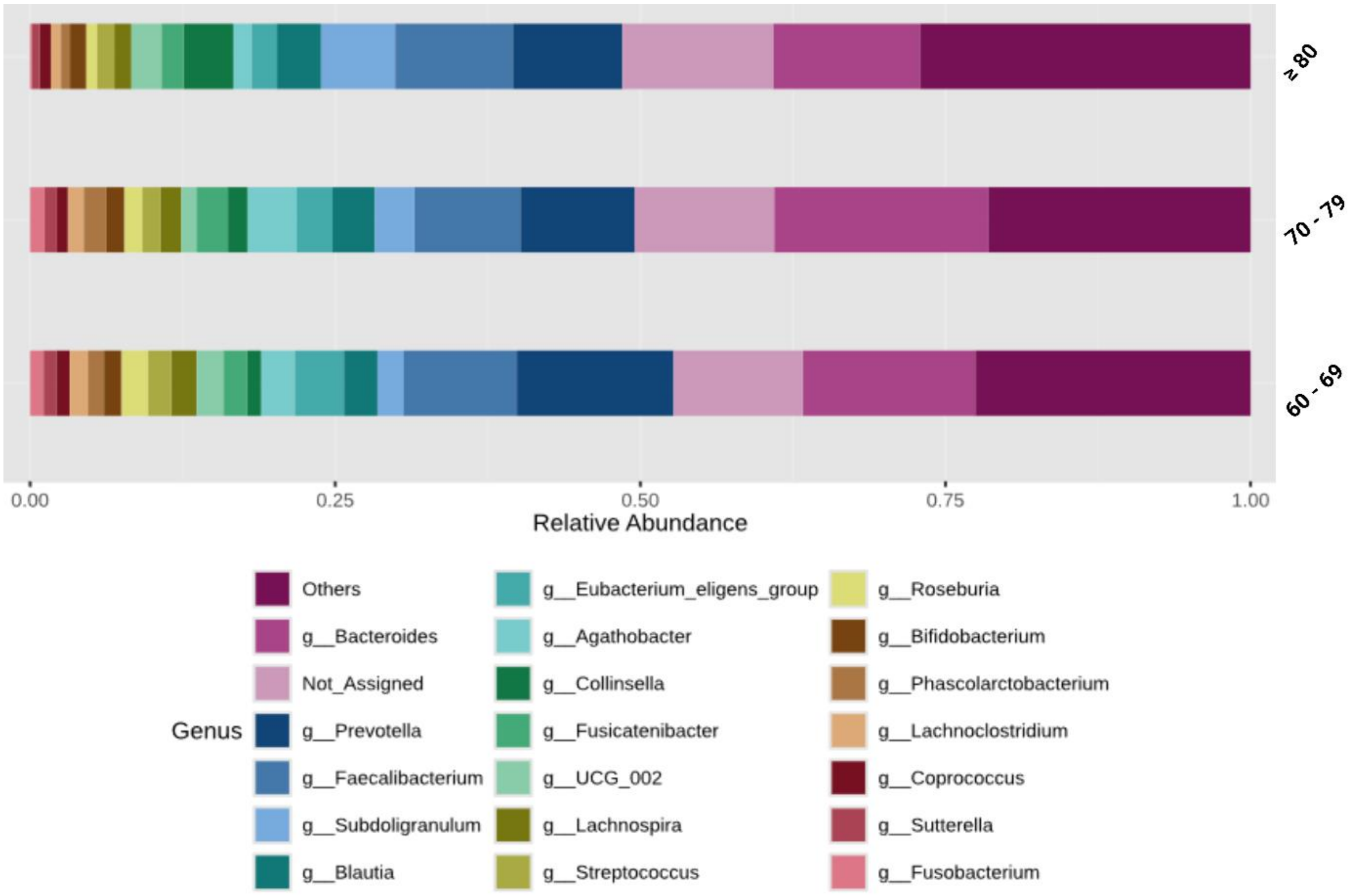

Figure S3. Genus-level composition of gut microbiota across different elderly age groups in both provinces. The stacked bar chart displays the relative abundance of the top 20 bacterial genera among three age groups: 60–69, 70–79, and ≥80 years.

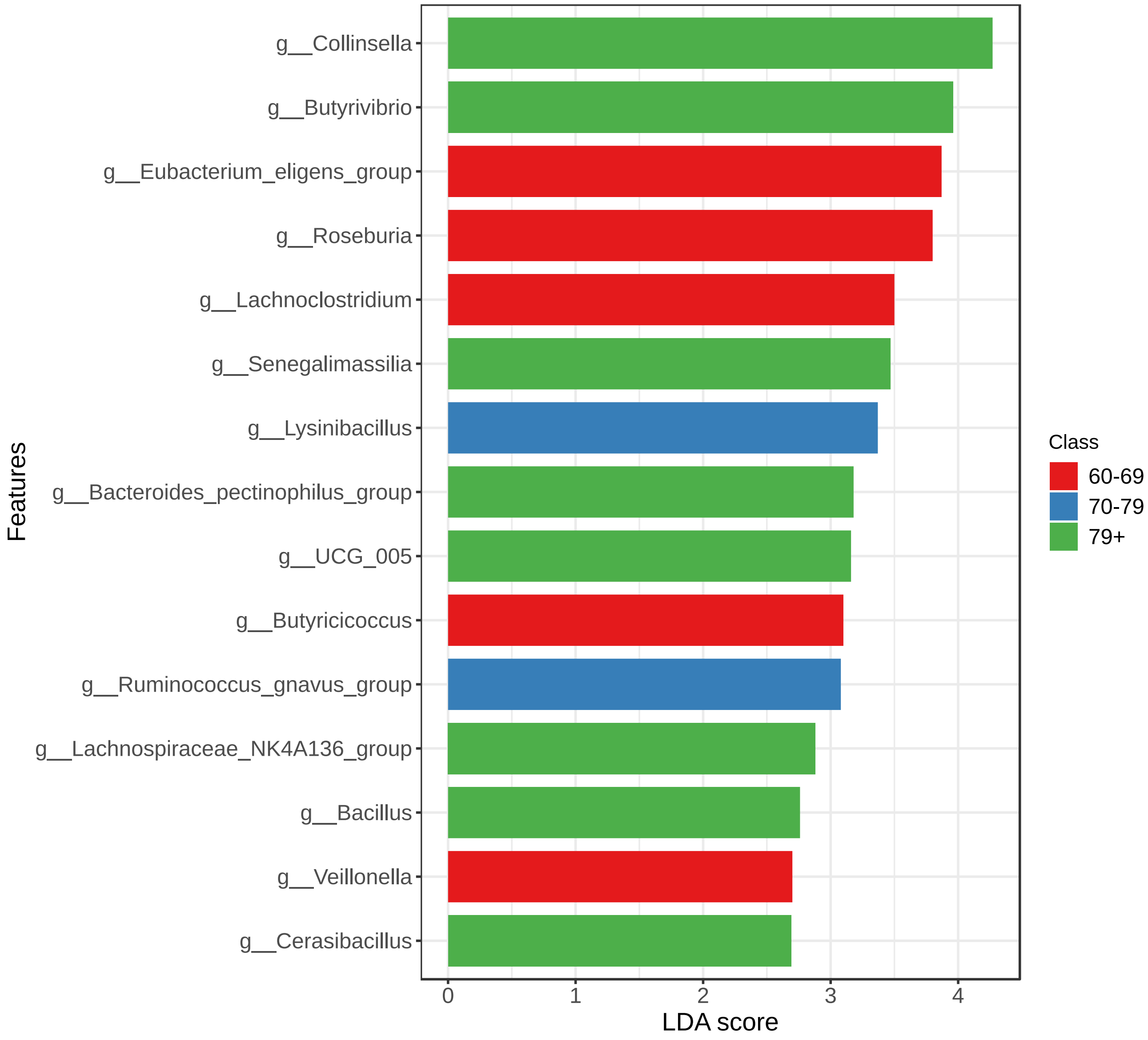

Figure S4. LEfSe analysis of differentially abundant genera across age groups (60–69 years, 70–79 years, and ≥80 years). Genera with LDA scores > 2.0 and $p < 0.05$ are shown, with colors indicating the age group in which each taxon was enriched.

Table S1: Statistical results for alpha diversity (Chao1 and Shannon indices) across regions and age groups.

Pair	Statistic	P-value	FDR	Diversity measure	Statistical method	Taxonomy level
U-Hanoi vs R-Hanoi	2037	0.019544	0.019544	Chao 1	Mann-Whitney	Genus
U-Hanoi vs R-Hanoi	1849	0.20175	0.20175	Shannon	Mann-Whitney	Genus
U-Thanh_Hoa vs R-Thanh_Hoa	1869	0.05255	0.05255	Chao 1	Mann-Whitney	Genus
U-Thanh_Hoa vs R-Thanh_Hoa	1438	0.54941	0.54941	Shannon	Mann-Whitney	Genus
70-79 vs 80+	911.5	0.002558	0.003837	Chao 1	Mann-Whitney	Genus
70-79 vs 60-69	2875	0.001305	0.003837	Chao 1	Mann-Whitney	Genus
80+ vs 60-69	2527.5	0.67058	0.67058	Chao 1	Mann-Whitney	Genus
70-79 vs 80+	1040	0.027019	0.040528	Shannon	Mann-Whitney	Genus
70-79 vs 60-69	3146	0.014381	0.040528	Shannon	Mann-Whitney	Genus
80+ vs 60-69	2425	0.98272	0.98272	Shannon	Mann-Whitney	Genus

Table S2: Statistical results for beta diversity (Bray–Curtis distances and PERMANOVA) across regions and age groups.

Pair	F- value	R-squared (R^2)	P-value	FDR	Ordination method	Distance method
U-Hanoi vs R-Hanoi	1.2636	0.010962	0.197	0.197	PCoA	Bray-Curtis Index
U-Thanh_Hoa vs R-Thanh_Hoa	6.295	0.054599	0.001	0.001	PCoA	Bray-Curtis Index
Age groups overall	2.5082	0.021904	0.001	0.003	PCoA	Bray-Curtis Index
70-79 vs 80+	2.4509	0.022393	0.007	0.0105	PCoA	Bray-Curtis Index
70-79 vs 60-69	1.5874	0.0085536	0.082	0.082	PCoA	Bray-Curtis Index
80+ vs 60-69	3.6458	0.022695	0.001	0.003	PCoA	Bray-Curtis Index

Table S3. Genus-level relative abundance (%) of dominant taxa in urban and rural Hanoi.

	U-Hanoi	R-Hanoi
g__Agathobacter	0.030256	0.044894
g__Akkermansia	0.01928	0.007967
g__Alistipes	0.010768	0.010921
g__Bacteroides	0.16057	0.172152
g__Bifidobacterium	0.033234	0.006873
g__Blautia	0.044208	0.026331
g__Christensenellaceae_R_7_group	0.015065	0.005942
g__Coprococcus	0.010741	0.009501
g__Eubacterium_eligens_group	0.042492	0.044728
g__Faecalibacterium	0.070857	0.074722
g__Fusicatenibacter	0.035319	0.01751
g__Lachnoclostridium	0.013867	0.0159
g__Lachnospira	0.020709	0.022198
g__Prevotella	0.073787	0.097626
g__Roseburia	0.017732	0.030665
g__Ruminococcus	0.011416	0.009544
g__Ruminococcus_torques_group	0.010002	0.012096
g__Streptococcus	0.013281	0.021794
g__Subdoligranulum	0.040322	0.021859
g__UCG_002	0.021586	0.021387
Not_Assigned	0.109094	0.117681
Others	0.195415	0.207708

Table S4. Genus-level relative abundance (%) of dominant taxa in urban and rural Thanh Hoa.

	U-Thanh_Hoa	R-Thanh_Hoa
g__Agathobacter	0.017387	0.022581
g__Bacteroides	0.054886	0.142682
g__Bifidobacterium	0.010083	0.010582
g__Blautia	0.021076	0.036125
g__Collinsella	0.061129	0.00994
g__Coprococcus	0.009406	0.014599
g__Eubacterium_eligens_group	0.021059	0.029729
g__Faecalibacterium	0.078126	0.083068
g__Fusicatenibacter	0.005498	0.031916
g__Fusobacterium	0.012566	0.018929
g__Lachnoclostridium	0.006544	0.017509
g__Lachnospira	0.010073	0.024916
g__Phascolarctobacterium	0.040306	0.011243
g__Prevotella	0.17448	0.10985
g__Roseburia	0.002886	0.01894
g__Streptococcus	0.01712	0.022795
g__Subdoligranulum	0.025388	0.039142
g__Sutterella	0.011666	0.009675
g__UCG_002	0.015855	0.023459
g__uncultured	0.009984	0.013367
Not_Assigned	0.132448	0.103388
Others	0.262033	0.205563

Table S5: Genus-level relative abundance (%) of dominant taxa across age groups (60–69, 70–79, and ≥79 years).

	**60-69**	**70-79**	**79+**
Not_Assigned	0.10598	0.114396	0.123523
Others	0.215615	0.205262	0.262808
g__Agathobacter	0.027674	0.0406	0.015149
g__Bacteroides	0.141267	0.175409	0.120403
g__Bifidobacterium	0.01451	0.014713	0.013561
g__Blautia	0.027499	0.034647	0.036301
g__Collinsella	0.011576	0.016107	0.040723
g__Coprococcus	0.01093	0.008837	0.009447
g__Eubacterium_eligens_group	0.040184	0.029301	0.020668
g__Faecalibacterium	0.092703	0.086952	0.096622
g__Fusicatenibacter	0.019335	0.025434	0.018215
g__Fusobacterium	0.011061	0.011935	0.001604
g__Lachnoclostridium	0.015185	0.013155	0.008131
g__Lachnospira	0.021153	0.017016	0.013763
g__Phascolarctobacterium	0.012814	0.018567	0.007296
g__Prevotella	0.128342	0.093451	0.089628
g__Roseburia	0.021837	0.014722	0.00896
g__Ruminococcus_torques_group	0.009884	0.009705	0.008082
g__Streptococcus	0.018742	0.014787	0.014128
g__Subdoligranulum	0.021671	0.032272	0.060358
g__Sutterella	0.010416	0.01014	0.006007
g__UCG_002	0.021619	0.012591	0.02462

Table S6. LEfSe-identified discriminative genera across age groups with corresponding LDA scores and relative abundance values.

	**P-values**	**FDR**	**60-69**	**70-79**	**80+**	**LDAscore**
g__Collinsella	0.000488	0.014331	13042	16536	49958	4.27
g__Butyrivibrio	0.000334	0.014331	523.7	0	18091	3.96
g__Eubacterium_eligens_group	0.000551	0.014331	35556	29953	20626	3.87
g__Roseburia	0.000827	0.016726	19129	12509	6409.4	3.8
g__Lachnoclostridium	0.000981	0.017263	14333	13734	7973.8	3.5
g__Senegalimassilia	6.59E-06	0.001199	330.92	329.23	6256.8	3.47
g__Lysinibacillus	0.004105	0.046692	496.36	5163.4	3805.7	3.37
g__Bacteroides_pectinophilus_group	0.000496	0.014331	1099.4	1009.5	4054.2	3.18
g__UCG_005	0.000657	0.014938	3110.2	2977	5837.8	3.16
g__Butyricicoccus	0.002192	0.030692	4404.4	3543.9	1895.7	3.1
g__Ruminococcus_gnavus_group	0.001859	0.028187	3927	4913.2	2492.3	3.08
g__Lachnospiraceae_NK4A136_group	0.002428	0.031566	6320.8	5349.9	6861.1	2.88
g__Bacillus	0.000141	0.008525	68.468	0	1161.6	2.76
g__Veillonella	0.003045	0.036943	1393.8	1291.5	386.22	2.7
g__Cerasibacillus	0.001043	0.017263	0	0	974.26	2.69

Table S7. Significant genus-level features from single-factor analysis comparing urban and rural Hanoi.

	**P-values**	**FDR**	**U-Hanoi**	**R-Hanoi**
g__Acinetobacter	0.00002831	0.003652	2107.1	18163
g__Bifidobacterium	0.0058749	0.039259	31656	12993.1
g__Blautia	0.0012546	0.012	46900	15450
g__Fusicatenibacter	0.008796	0.048759	34582	18756
g__Collinsella	0.0070245	0.042251	15020.8	7095.6
g__Fusobacterium	0.007952	0.046528	1528.7	8259.5

Table S8. Significant genus-level features from single-factor analysis comparing urban and rural Thanh Hoa.

	Pvalues	FDR	U-Thanh_Hoa	R-Thanh_Hoa
g__Collinsella	1.82E-08	2.64E-06	63138	12859
g__Fusicatenibacter	1.91E-07	9.21E-06	6646.7	34512
g__Roseburia	3.55E-06	0.000129	2687.5	17067
g__Bacteroides	6.98E-06	0.000202	58184	168000
g__Lachnospira	0.00567	0.039149	8662.1	21104
g__Blautia	0.007419	0.039843	19709	35119

Table S9. Significant genus-level trends from single-factor analysis across age groups (60–69, 70–79, and ≥79 years).

	**P-values**	**FDR**	**60-69**	**70-79**	**79+**
g__Senegalimassilia	6.59E-06	0.001199	330.92	329.23	6256.8
g__Collinsella	0.000488	0.014331	13042	16536	49958
g__Roseburia	0.000827	0.016726	19129	12509	6409.4
g__Veillonella	0.003045	0.036943	1393.8	1291.5	386.22
